# Beliefs, behaviours, and attitudes towards tanning and melanoma in the Irish population

**DOI:** 10.1002/ski2.398

**Published:** 2024-05-30

**Authors:** Catriona Gallagher, Cathal O’Connor, Eimear Gilhooley, John Bourke, Michelle Murphy

**Affiliations:** ^1^ Dermatology South Infirmary Victoria University Hospital Cork Ireland; ^2^ Medicine University College Cork Cork Ireland; ^3^ INFANT Research Centre University College Cork Cork Ireland

## Abstract

**Background:**

The incidence of melanoma continues to rise in Ireland. Skin cancer prevention campaigns rely on promoting knowledge to improve sun‐related behaviour.

**Objectives:**

To explore beliefs, behaviours, and attitudes towards tanning, and confidence in identifying signs of melanoma in the Irish population.

**Methods:**

A cross‐sectional study was performed via an online questionnaire, with questions related to tanning, sun exposure, and skin cancer behaviours. Respondents were recruited according to gender, age and geographic region.

**Results:**

The questionnaire was completed by 1043 respondents (response rate 85%). Mean age was 41 years (range 20–72 years). Participants had mixed awareness of risk reduction strategies for melanoma but had high perceived concerns about developing melanoma. However, 48.9% regularly sunbathed when sunny in Ireland and 41.5% had used tanning beds. The most common reason for not photoprotecting while sunbathing was because it prevented tanning. Nearly half (45.9%) of those who sunbathed agreed that it was worth getting sunburned to get a tan, and 69.4% reported feeling and looking better with a tan. Less than half (42.4%) felt confident about what to look for when checking their skin for melanoma.

**Conclusions:**

This study underscores the importance of addressing the cultural and aesthetic aspects of sun‐tanning behaviour in skin cancer prevention efforts, as well as increasing awareness of skin cancer signs and self‐examination. Further research into the potential addictive nature of UV‐seeking behaviour may offer new avenues for intervention and support for individuals who are addicted to tanning.

**What is already known?**

- The incidence of melanoma continues to rise, and public education on the risks of ultraviolet radiation is a key focus of skin cancer prevention campaigns.
- The public are aware of sun protection measures but less knowledgeable on signs of skin cancer and how to perform self‐skin examination.
- Public awareness of skin cancer risks does not always lead to improved sun protection measures.

**What does this study add?**

- This study highlights that tanning and a tanned appearance remain appealing in Ireland and that tanning addiction may be common.
- Concerningly, respondents were not confident in their ability to check their own skin for melanoma.
- Public health campaigns require a multifaceted approach to shift society's perception of tanned skin as a paragon of beauty, and to enhance self‐examination and identification of signs of skin cancer.

## 1 INTRODUCTION

Melanoma is on the rise worldwide, with nearly 300 000 new cases and over 60 000 deaths attributed to it annually.^1^ In Ireland, the incidence of melanoma has increased by 81% since 1994, with over 1100 new cases per year.^2^ It accounts for 2% of all cancer‐related deaths annually in Ireland.^2^ Ultraviolet (UV) radiation is classified as a Group 1 carcinogen by the International Agency for Research on Cancer and is the main exogenous risk factor for melanoma.^3^ In particular, intermittent sun exposure and sunburn, especially in early life, confer an increased risk of melanoma.^4^ In addition, the native Irish population has the most lightly pigmented skin in the world,^5^ dominated by Fitzpatrick skin types 1 and 2, placing this population at increased susceptibility to melanoma.^6^ Given the rising incidence, and morbidity, mortality, and economic costs associated with melanoma, the Irish government has focussed on skin cancer prevention campaigns. Skin cancer prevention campaigns are based on the hypothesis that improved knowledge about the importance of photoprotection will lead to changes in sun‐related behaviours. However, public awareness of skin cancer risks does not always translate to improved sun protective measures.^7^ Previous research in the United Kingdom (UK) has shown that, although 92% of participants knew that excessive UV exposure causes skin cancer and 100% knew that sunburn was unhealthy, the majority also thought that tanned skin was attractive (80%) and that tanned people looked healthier (71%).^8^ Norway, like Ireland, has a high rate of melanoma despite limited natural sun exposure in a lightly pigmented populace, indicating that tanning behaviour is a significant contributor to skin cancer risk. Previous research has assessed tanning behaviour in the Norwegian population using a modified Health Belief Model, based on the hypothesis that increased knowledge about beliefs, attitudes and sun‐tanning behaviour can provide information to inform future sun protection interventions.^9^ To our knowledge, this research has not been replicated in an Irish population. The aim of this study was to explore behaviour towards photoprotection, attitudes towards tanning, and awareness of signs of melanoma in the Irish population.

## 2 MATERIALS AND METHODS

### 2.1 Study design

A cross‐sectional study was performed by collecting data through an online survey in December 2020. Data collection was assisted by a market research company called Real Insights, who provided an online consumer panel. Proportions were set for age, gender and geographic region within Ireland, to be nationally representative. Participants were included/excluded based on their answers to these questions. Patients with a previous personal history of melanoma or other skin cancer were excluded from the study.

### 2.2 Questionnaire

The self‐reported questionnaire was adapted from the previously validated questionnaire used in the Norwegian study,^8^ with permission from the authors. It consisted of 43 questions in total and took approximately 15 min to complete. The focus of the survey was perceived behavioural risk factors for melanoma and motivational/attitudinal factors to tanning.

### 2.3 Statistics

Demographic and clinical data were summarised using descriptive statistics (SPSS version 24). MedCalc Software Ltd (2021) was used to calculate the odds ratio (OR), standard error and 95% confidence interval (CI). A *p* value <0.05 was considered statistically significant.

## 3 RESULTS

### 3.1 Participants

A total of 1227 individuals were invited to participate, and 1043 respondents completed the questionnaire (Table 1); giving a response rate of 85%. Just over half (52%, *n* = 542) were female, and the mean age was 41 years (range 20–72 years). The geographical spread of respondents was representative of the Irish population: Dublin 29.6% (*n* = 309), Leinster excluding Dublin 23.9% (*n* = 249), Munster 27.5% (*n* = 287) and Connaught/Ulster 19% (*n* = 198). Half (49.9%, *n* = 521) completed a bachelor's degree or higher, in keeping with the high levels of third level education in Irish adults.^10^ One fifth (20.3%, *n* = 211) had at least one family member with a history of melanoma and almost one third (29.2%, *n* = 294) had an acquaintance who had previously had melanoma.

**TABLE 1:** Participant demographics and risk factors for skin cancer.

Background/demographics	*n* (%)
Gender	
Male	501 (48)
Female	542 (52)
Age	
Mean	41 years
Range	20–72 years
Geographic region	
Dublin	309 (29.6)
Leinster outside of Dublin	249 (23.9)
Munster	287 (27.5)
Connaught‐Ulster	198 (19)
Educational level	
Lower secondary or lower	96 (9.2)
Upper secondary to certificate	426 (40.8)
Bachelor degree/diploma	354 (33.9)
Masters or PhD	167 (16)
Do you have a family history of melanoma?	
No, none in my family	763 (73.2)
Yes, one family member	179 (17.2)
Yes, several family members	32 (3.1)
I do not know/uncertain	69 (6.6)
Do you know anyone who has had a melanoma?	
No, none in my circle of acquaintances	636 (61)
Yes, one	254 (24.4)
Yes, several	50 (4.8)
I do not know/uncertain	103 (9.9)

### 3.2 Knowledge of the role of sun protection and risk of sunbathing

Participants had mixed knowledge of the beneficial role of sun protection but were aware of the risks and dangers associated with sunbathing and melanoma (Table 2). For example, only three out of five respondents knew that sun protection factor (SPF) of 15 or more reduced the risk of sunburn (57.6%) and melanoma (63.6%). Less than three in four knew that staying in the shade reduced the risk of sunburn (73.9%) and melanoma (68.1%). Just over half (55.1%) knew that avoiding the sun during the middle of the day reduced the risk of sunburn, and two‐thirds (68.4%) knew that covering up with clothing in the sun reduced the risk of melanoma. Conversely, 82.9% believed that it was important to avoid getting melanoma, 78.8% believed that developing melanoma would have serious consequences, and 73.4% believed that getting sunburned was serious. Almost a quarter (23.9%) believed that it was easy to treat melanoma.

**TABLE 2:** Knowledge of the role of sun protection and risk of sunbathing (*n* = 1043).

	Agree *n* (%)
Sun protection knowledge	
Using sun protection prevents me from getting a tan	305 (29.2)
Using sunscreen with a sun protection factor (SPF) of 15 or more reduces the risk of sunburn	601 (57.6)
Using sunscreen with SPF 15 or more reduces the risk of getting melanoma	663 (63.6)
Staying in the shade reduces the risk of sunburn	771 (73.9)
Staying in the shade reduces the risk of getting melanoma	720 (68.1)
Avoiding the sun between 11 AM and 3 PM reduces the risk of being sunburned.	575 (55.1)
Covering up with clothes when in the sun reduces the risk of getting melanoma	713 (68.4)
Perceived risk and consequences of sunbathing	
I believe that getting sunburned is serious	766 (73.4)
Developing melanoma would have serious consequences for me	822 (78.8)
It is important for me to prevent getting melanoma	863 (82.7)
Developing melanoma in the future worries me	646 (61.9)
I think it is easy to treat melanoma	275 (23.7)

### 3.3 Sun behaviour

Over two in five (41.5%) participants had ever used a sunbed (Table 3), with 10.6% (*n* = 111) having used a sunbed in the previous year, with an average of eight sessions in the last year (range 1–100). Over two in five (40.8%) had spent at least 2 weeks on sun holiday in 2019. Almost half (48.9%) sought the sun to sunbathe. For those who sunbathed (*n* = 510), the motivating reasons for sunbathing included vitamin D supply (70.8%), feeling/looking better with tanned skin (69.4%), finding it comfortable (62.2%), socialising with friends/family (52.4%), and needing a tan for their job/hobby (24.5%). For those who did not sunbathe (*n* = 533), the motivating reasons for avoiding the sun included not wanting to get sunburned (79%), finding it uncomfortable (66.2%), concern about wrinkles/pigmentation (65.3%), concern about skin cancer (64.4%), and not having time to sunbathe (60.2%). Only 35.3% of those who did not sunbathe did not want to get a tan, and only 17.6% found tanned skin unattractive. Of the sunbathing group (*n* = 510), 45.9% felt that it was worth getting slightly sunburned to get a tan. Of sunbathers (*n* = 510), less than half (47.9%) always or often wore sunscreen, and only one in three aways or often covered up in clothing (34.5%) or took breaks from the sun in shade or indoors (37.5%) (Table 4). Significant proportions of those who sunbathed felt that sunscreen was inconvenient (44.9%), easy to forget (42.9%), unpleasant to apply (38%), prevented tanning (37.6%), expensive (34.3%), uncomfortable (32.9%), and time‐consuming (32.3%). Sunbathers reported that covering up with clothes prevented tanning (48%), was uncomfortable (46.3%), unpleasant (42.5%), inconvenient (42%), or embarrassing (25.7%). Sunbathers reported that taking breaks from sunbathing prevented tanning (36.9%) and was inconvenient (35.9%).

**TABLE 3:** Responses to questions related to sun behaviour.

Sun behaviours	*n* (%)
Have you used a sunbed in last 12 months?	
Yes	111 (10.6)
No (but I have previously used a sunbed)	322 (30.9)
I have never used a sunbed in my life	610 (58.5)
How many weeks did you spend on sun holidays in 2019?	
None	306 (29.3)
1 week or less	312 (29.9)
2–3 weeks	341 (32.7)
4 weeks or more	84 (8.1)
Do you seek the sun to sunbathe?	
Yes	510 (48.9)
No	533 (51.1)
If you seek the sun to sunbathe, what are your reasons for doing so? (*n* = 510)	
I want a supply of vitamin D	361 (70.8)
I feel or look better with tanned skin	354 (69.4)
I find it comfortable	317 (62.2)
It is social to sunbathe with friends/family	267 (52.4)
My friends sunbathe and have a tanned skin	231 (45.3)
I need a tan for my job, sport activity or hobby	125 (24.5)
If you do not seek the sun to sunbathe, what are your reasons for avoiding the sun? (*n* = 533)	
Do not want to get sunburned	421 (79)
Find sunbathing boring or uncomfortable	353 (66.2)
Are concerned about getting wrinkles or pigmentary changes	348 (65.3)
Are afraid of getting skin cancer	343 (64.4)
Do not have time to sunbathe	321 (60.2)
Do not want to get a tan	188 (35.3)
Do not have friends or family who sunbathe	152 (28.5)
Find tanned skin unattractive	94 (17.6)

**TABLE 4:** Photoprotection frequency and reported reasons for not practicing photoprotection in those who seek the sun to sunbathe (*n* = 510).

If you seek the sun to sunbathe, do you wear sunscreen while sunbathing?	
Always (approximately 90%–100% of the time)	84 (16.5)
Often (approximately 60%–80% of the time)	160 (31.4)
Sometimes (approximately 30%–50% of the time)	143 (28)
Seldom (approximately 5%–20% of the time)	82 (16.1)
Never	41 (8)
If you seek the sun to sunbathe, do you find sunscreen	
Inconvenient	229 (44.9)
Easy to forget	219 (42.9)
Unpleasant to apply	194 (38)
Prevents you getting a tan	192 (37.6)
Expensive	175 (34.3)
Uncomfortable	168 (32.9)
Time‐consuming	165 (32.3)
If you seek the sun to sunbathe, do you cover up with clothing to avoid the sun while sunbathing?	
Always (approximately 90%–100% of the time)	51 (10)
Often (approximately 60%–70% of the time)	125 (24.5)
Sometimes (approximately 30%–50% of the time)	183 (35.9)
Seldom (approximately 10%–20% of the time)	118 (23.1)
Never	33 (6.5)
If you seek the sun to sunbathe, do you find covering up with clothes	
Prevents you getting a tan	245 (48)
Uncomfortable	236 (46.3)
Unpleasant to do	217 (42.5)
Inconvenient	214 (42)
Embarrassing	131 (25.7)
If you seek the sun to sunbathe, do you take breaks (in the shadow or indoors) to avoid the sun?	
Always (90%–100% of the time)	68 (13.3)
Often (60%–70% of the time)	125 (24.5)
Sometimes (30%–50% of the time)	180 (35.3)
Seldom (10%–20% of the time)	101 (19.8)
Never	36 (7.1)
If you seek the sun to sunbathe, do you find taking breaks from the sun	
Prevents you getting a tan	188 (36.9)
Inconvenient	183 (35.9)

### 3.4 Melanoma risk management

Two‐thirds of respondents felt that they had control over whether they developed skin cancer in future (64.3%) and that they would photoprotect even if those around them did not (69.1%). Four in five (79.3%) felt that they knew what to do to avoid getting sunburned. However, only two in five felt that they were able to detect warning signs of melanoma at an early stage (39.8%) and that they were confident in what to look for when checking their skin for melanoma (42.4%) (Table 5).

**TABLE 5:** Confidence in control and assessment of risk of melanoma (*n* = 1043).

Perceived control and influence over own life	Agree *n* (%)
I feel I have control about whether I get skin cancer in the future	671 (64.3%)
I am certain that I will protect myself against the sun, even if those accompanying me do not	721 (69.1%)
I feel I know what to do to avoid getting sunburned	807 (79.3%)
I am able to detect warning signs of melanoma at an early stage	415 (39.8%)
I feel confident about what to look for when checking my skin for warning signs of melanoma	442 (42.4%)

### 3.5 Subgroup analysis

Risk factors for sun‐seeking behaviour were also explored by dichotomising the respondents into two groups—those who sunbathed to get a tan and those who did not sunbathe (Table S1). Respondents who were more likely to sunbathe were 18–24 years old (OR 1.77, 95% CI 1.19–2.62, *p* < 0.01), 35–44 years old (OR 1.4, 95% CI 1.04–1.89, *p* = 0.03), know someone who had a history of melanoma (OR 1.53, 95% CI 1.16–2.00, *p* < 0.01), have previously used a sunbed (OR 1.6, 95% CI 1.26–2.03, *p* < 0.001), feel confident in their ability to detect melanoma early (OR 1.38, 95% CI 1.08–1.77, *p* = 0.01), or feel confident about what to look for when self‐examining their skin (OR 1.29, 95% CI 1.01–1.65, *p* < 0.05). Respondents who were less likely to sunbathe were 55 years or older (OR 0.52, 95% CI 0.4–0.68, *p* < 0.0001), were afraid of getting skin cancer (OR 0.64, 95% CI 0.44–0.92, *p* = 0.01), and did not want to get wrinkles or pigmentation (OR 0.65, 95% CI 0.45–0.94, *p* = 0.02). There was no significant difference in sunbathing behaviours according to gender, education levels, or geographical locations.

## 4 DISCUSSION

The findings from this large survey study provide valuable insights into tanning behaviour in the Irish population and its implications for skin cancer prevention. It is believed that by improving public awareness of skin cancer, photoprotection and sun avoidance, the trajectory of skin cancer incidence can be reduced.^11^ However, it has long been known that good health literacy does not directly correlate with better lifestyle behaviours.^7^ Our research found an obvious disconnect between participants' beliefs and their behaviour. Although the majority were aware of the risks associated with UV exposure, they were still willing to take those risks to achieve a tan and had poor knowledge about the early detection of melanoma. This is also in keeping with previous research showing that public awareness of skin cancer risks does not always lead to improved sun protection measures.^12^

The majority of respondents considered tanned skin to be attractive; whether this popularity of bronzed skin represents a biological addiction or an aesthetic preference remains incompletely understood.^13^ The glamourization of tanning in Europe and North America can be traced back over a century and has been perpetuated through media, fashion, and societal norms, with tanned skin being associated with status and attractiveness. Despite the growing awareness of the dangers of tanning and the link between UV radiation and skin cancer, changing these deeply ingrained cultural and social beliefs remains a significant challenge for melanoma prevention efforts. Public health campaigns need a multifaceted approach to shift the public's perception of tanned skin as the epitome of beauty, redefine beauty standards and promote skin health. This will likely require collaboration with multiple stakeholders, including media, education, community organisations, and influencers. Furthermore, research in mouse studies suggests that repeated UV exposure can lead to an addiction‐like response. This is thought to be mediated by elevated levels of β‐endorphin, a natural opioid peptide, leading to increased nociceptive thresholds that are reversed by naloxone.^13^ There is also evidence that supports the notion of tanning addiction in humans. Some UV‐seekers meet criteria for substance‐related disorders according to CAGE and DSM‐IV criteria,^14^ suggesting that their tanning behaviour may share similarities with addiction to substances like drugs or alcohol. Other studies have shown that UV‐seekers are capable of distinguishing between actual UV exposure and mock treatment in blind tanning bed experiments indicating that UV is a reinforcing stimulus, with subjects finding it desirable and feeling a sensation of greater relaxation.^15^ Moreover, opioid blockade has been shown to reduce UV preference in frequent tanners, as well as withdrawal‐like symptoms with naltrexone administration^16^ While further research is needed, this does support the possibility that some individuals may be addicted to tanning.

There were some interesting associations between the beliefs of those who sunbathed regarding sunbathing and melanoma risks. Respondents who sunbathed were more likely to know of someone who had been diagnosed with melanoma, suggesting that familiarity with melanoma does not reduce behaviours that increase the risk of developing it. They were also more likely to have used a sunbed, to not be afraid of getting wrinkles or skin pigmentation, and to not be afraid of getting skin cancer, which is unsurprising as sunbathing and sunbeds are both well known to increase the risk of premature ageing and skin cancer. Counterintuitively, participants who sunbathed were more confident in their ability to detect melanoma early and in self‐examining their skin, which may represent a form of cognitive dissonance. Individuals who sunbathe may be more likely to seek reassurance and minimise the risk of sun exposure and skin cancer, and may be more susceptible to misinformation related to these topics.^17^ The high rates of sunbed use in this study (40%) is extremely concerning given the dramatically increased risk of skin cancer associated with their use. This study found that those ages 18–24 and 35–44 were more likely to sunbathe, while those 25–34 were not significantly more likely to sunbathe. This age group has been associated with a propensity for tanning previously. There may have been insufficient respondents in this age group to achieve statistically significant results.

Comparison to the Norwegian study (*n* = 569)^9^ provide some insights into regional variations in sun‐related behaviours, highlighting differences in sunbed usage, sunscreen compliance and shade‐seeking. While the clinical data might not have been directly comparable due to the use of a modified Health Belief Model in the Norwegian study, a key similarity emerged; participants in both regions felt and looked better with a tan (69% and 85%). This commonality highlights the prevailing attraction of tanned skin which is common in lightly pigmented populations in Northern Europe, and emphasises the significant challenge that public health campaigns face in changing this deeply ingrained perception. While more Norwegians reported using sunbeds in the previous year (16% vs. 10%), this survey was performed during the Covid‐19 pandemic when tanning salons were closed as non‐essential businesses. Similar to data from the UK,^8^ 82% of participants found tanned skin attractive, despite knowing that UV exposure directly causes skin cancer.

Additionally, this study found that confidence in identifying signs of skin cancer and knowledge in how to perform self‐skin examination was low among study participants. This lack of awareness is consistent with previous research,^11^ and highlights a clear need for targeted public health campaigns that focus on the visual physical signs that could indicate skin cancer, resulting in early detection, better outcomes and reduced mortality rates.

This study benefits from a large sample size and excellent representativeness of the Irish population, reducing the potential for selection bias, making the findings more applicable to the broader population. Achieving a response rate of 85% helps to ensure that the data collected are more representative and less prone to non‐response bias. It is limited by the reliance on self‐reported retrospective data which introduces the potential for recall bias and imprecisions. The study was performed in December 2020. Completing this study in a dark winter month may yield different responses to completion in the summer. There may also have been changes in attitudes during or since the Covid‐19 pandemic which may not have been captured.

In summary, this study underscores the importance of addressing not only knowledge but also the cultural and addictive aspects of sun‐tanning behaviour in skin cancer prevention efforts. Public health campaigns that focus on changing attitudes towards tanning and increasing awareness of skin cancer signs and self‐examinations can play a crucial role in reducing skin cancer rates and improving outcomes. Additionally, further research into the potential addictive nature of UV‐seeking behaviour may offer new avenues for intervention and support for individuals who may be addicted to tanning.

## CONFLICT OF INTEREST STATEMENT

The authors declare that they have no conflicts of interest.

## AUTHOR CONTRIBUTIONS

**Catriona Gallagher**: Conceptualization (equal); data curation (equal); formal analysis (equal); funding acquisition (equal); investigation (equal); methodology (equal); project administration (equal); resources (equal); software (equal); supervision (equal); validation (equal); visualization (equal); writing – original draft (equal); writing – review & editing (equal). **Cathal O'Connor**: Data curation (equal); formal analysis (equal); investigation (equal); methodology (equal); project administration (equal); resources (equal); software (equal); supervision (equal); validation (equal); visualization (equal); writing – original draft (equal); writing – review & editing (equal). **Eimear Gilhooley**: Conceptualization (equal); data curation (equal); formal analysis (equal); investigation (equal); project administration (equal); supervision (equal); validation (equal); writing – review & editing (equal). **John Bourke**: Conceptualization (equal); formal analysis (equal); funding acquisition (equal); investigation (equal); methodology (equal); project administration (equal); resources (equal); software (equal); supervision (equal); validation (equal); visualization (equal); writing – review & editing (equal). **Michelle Murphy**: Conceptualization (equal); data curation (equal); formal analysis (equal); funding acquisition (equal); investigation (equal); methodology (equal); project administration (equal); resources (equal); software (equal); supervision (equal); validation (equal); visualization (equal); writing – review & editing (equal).

## ETHICS STATEMENT

The local ethics committee deemed that ethical approval was not required for collection, analysis, and publication of data from this anonymous non‐interventional survey study.

## Supporting information

Table S1

## Data Availability

The data underlying this article will be shared on reasonable request to the corresponding author.
