# Supplementary material for: Beliefs, behaviours, and attitudes towards tanning and melanoma in the Irish population
Source: Skin Health Dis. 2024 May 30;4(5):e398. doi: 10.1002/ski2.398 (PMC11442063; doi:10.1002/ski2.398)
Supplement: Supplementary file 1 — Table S1 [file SKI2-4-e398-s001.docx]

Supplementary Table 1. Subgroup Analysis – risk factors for sun-seeking behaviour according to those who sunbathed to get a tan and those who did not sunbathe.

|  | P value |
| --- | --- |
| Male vs female | \| Odds ratio \| 0.9991 \| \| --- \| --- \| \| 95 % CI: \| 0.7833 to 1.2744 \| \| z statistic \| 0.007 \| \| Significance \| P = 0.9943 \| |
| Age |  |
| 18-24 | \| Odds ratio \| 1.7695 \| \| --- \| --- \| \| 95 % CI: \| 1.1936 to 2.6231 \| \| z statistic \| 2.841 \| \| Significance \| **P = 0.0045** \| |
| 25-34 | \| Odds ratio \| 1.1112 \| \| --- \| --- \| \| 95 % CI: \| 0.8116 to 1.5213 \| \| z statistic \| 0.658 \| \| Significance \| P = 0.5107 \| |
| 35-44 | \| Odds ratio \| 1.4023 \| \| --- \| --- \| \| 95 % CI: \| 1.0395 to 1.8918 \| \| z statistic \| 2.214 \| \| Significance \| **P = 0.0269** \| |
| 45-54 | \| Odds ratio \| 1.0930 \| \| --- \| --- \| \| 95 % CI: \| 0.7946 to 1.5033 \| \| z statistic \| 0.547 \| \| Significance \| P = 0.5846 \| |
| 55+ | \| Odds ratio \| 0.5172 \| \| --- \| --- \| \| 95 % CI: \| 0.3956 to 0.6762 \| \| z statistic \| 4.820 \| \| Significance \| **P < 0.0001** \| |
| Dublin | \| Odds ratio \| 1.1495 \| \| --- \| --- \| \| 95 % CI: \| 0.8779 to 1.5052 \| \| z statistic \| 1.013 \| \| Significance \| P = 0.3111 \| |
| Rest of Leinster | \| Odds ratio \| 0.9974 \| \| --- \| --- \| \| 95 % CI: \| 0.7584 to 1.3118 \| \| z statistic \| 0.018 \| \| Significance \| P = 0.9854 \| |
| Munster | \| Odds ratio \| 1.0885 \| \| --- \| --- \| \| 95 % CI: \| 0.8276 to 1.4315 \| \| z statistic \| 0.606 \| \| Significance \| P = 0.5443 \| |
| Connaught/Ulster | \| Odds ratio \| 0.7450 \| \| --- \| --- \| \| 95 % CI: \| 0.5396 to 1.0287 \| \| z statistic \| 1.788 \| \| Significance \| P = 0.0737 \| |
| Ordinary Bachelor Degree or National Diploma NFQ Level 7 or above | \| Odds ratio \| 1.1866 \| \| --- \| --- \| \| 95 % CI: \| 0.9305 to 1.5131 \| \| z statistic \| 1.379 \| \| Significance \| P = 0.1678 \| |
| Have you or any of your biological family ever been diagnosed with skin cancer- melanoma? | \| Odds ratio \| 1.2634 \| \| --- \| --- \| \| 95 % CI: \| 0.9333 to 1.7103 \| \| z statistic \| 1.513 \| \| Significance \| P = 0.1302 \| |
| Has anyone in your circle of acquaintances been diagnosed with melanoma? | \| Odds ratio \| 1.5254 \| \| --- \| --- \| \| 95 % CI: \| 1.1584 to 2.0087 \| \| z statistic \| 3.007 \| \| Significance \| **P = 0.0026** \| |
| If you sunbathe in the beginning of the summer without using sun protection does your skin go Brown-tanned without first getting red | \| Odds ratio \| 1.2200 \| \| --- \| --- \| \| 95 % CI: \| 0.9376 to 1.5875 \| \| z statistic \| 1.481 \| \| Significance \| P = 0.1387 \| |
| Have you used a sunbed | \| Odds ratio \| 1.5992 \| \| --- \| --- \| \| 95 % CI: \| 1.2576 to 2.0335 \| \| z statistic \| 3.830 \| \| Significance \| **P = 0.0001** \| |
| It is likely I could develop melanoma during my lifetime | \| Odds ratio \| 0.7618 \| \| --- \| --- \| \| 95 % CI: \| 0.5755 to 1.0084 \| \| z statistic \| 1.902 \| \| Significance \| P = 0.0572 \| |
| Developing melanoma would have serious consequences for me | \| Odds ratio \| 1.1344 \| \| --- \| --- \| \| 95 % CI: \| 0.7706 to 1.6699 \| \| z statistic \| 0.639 \| \| Significance \| P = 0.5228 \| |
| Developing melanoma in the future worries me | \| Odds ratio \| 1.2501 \| \| --- \| --- \| \| 95 % CI: \| 0.9060 to 1.7251 \| \| z statistic \| 1.359 \| \| Significance \| P = 0.1742 \| |
| I think it is easy to treat melanoma | \| Odds ratio \| 1.1642 \| \| --- \| --- \| \| 95 % CI: \| 0.8610 to 1.5741 \| \| z statistic \| 0.988 \| \| Significance \| P = 0.3233 \| |
| I am able to detect warning signs of melanoma at an early stage | \| Odds ratio \| 1.3800 \| \| --- \| --- \| \| 95 % CI: \| 1.0761 to 1.7698 \| \| z statistic \| 2.538 \| \| Significance \| **P = 0.0112** \| |
| I feel confident about what to look for when checking my skin for warning signs of melanoma | \| Odds ratio \| 1.2897 \| \| --- \| --- \| \| 95 % CI: \| 1.0082 to 1.6499 \| \| z statistic \| 2.025 \| \| Significance \| **P = 0.0429** \| |
| I'm afraid of getting skin cancer | \| Odds ratio \| 0.6377 \| \| --- \| --- \| \| 95 % CI: \| 0.4442 to 0.9154 \| \| z statistic \| 2.439 \| \| Significance \| **P = 0.0147** \| |
| I do not want to get wrinkles or pigmentations in the skin | \| Odds ratio \| 0.6495 \| \| --- \| --- \| \| 95 % CI: \| 0.4501 to 0.9371 \| \| z statistic \| 2.307 \| \| Significance \| **P = 0.0210** \| |
| I believe getting a sunburn is serious | \| Odds ratio \| 0.7097 \| \| --- \| --- \| \| 95 % CI: \| 0.5025 to 1.0023 \| \| z statistic \| 1.947 \| \| Significance \| P = 0.0516 \| |
